# Anesthesia Management in Antenatal Care for Women With Chronic Kidney Disease: A Comprehensive Review

**DOI:** 10.7759/cureus.66389

**Published:** 2024-08-07

**Authors:** Angan Ghosh, Sanjot Ninave

**Affiliations:** 1 Anesthesiology, Jawaharlal Nehru Medical College, Datta Meghe Institute of Higher Education and Research, Wardha, IND

**Keywords:** fetal health, maternal outcomes, perioperative care, anesthesia management, pregnancy, chronic kidney disease

## Abstract

Chronic kidney disease (CKD) presents significant challenges in the management of pregnant women due to its impact on renal function and cardiovascular stability. This review examines the crucial role of anesthesia management in antenatal care for women with CKD, focusing on the complexities introduced by renal dysfunction and the implications for maternal and fetal health outcomes. The review discusses the physiological changes in CKD during pregnancy, highlighting the increased risks of hypertension, proteinuria, and adverse fetal outcomes. Anesthesia considerations, including the choice of anesthesia techniques (general anesthesia, regional anesthesia), perioperative monitoring, and management of fluid and electrolyte balance, are analyzed in the context of CKD-specific challenges. Clinical outcomes and current evidence regarding anesthesia efficacy and safety in CKD patients are reviewed, emphasizing the need for tailored anesthesia protocols to ensure optimal maternal comfort and fetal safety. The review concludes by identifying research gaps and proposing future directions to enhance anesthesia practices and improve outcomes for pregnant women with CKD undergoing surgical interventions or labor management.

## Introduction and background

Chronic kidney disease (CKD) presents unique challenges in the context of pregnancy, affecting approximately 3% of pregnancies globally. CKD is characterized by a gradual decline in kidney function over time, often accompanied by complications such as hypertension and proteinuria [1]. These physiological changes can complicate pregnancy by increasing the risk of adverse maternal outcomes such as worsening renal function, hypertensive disorders like preeclampsia, and preterm birth. The altered renal physiology during pregnancy, including increased glomerular filtration rate and changes in renal blood flow, further exacerbates these challenges. Consequently, managing CKD during pregnancy requires a multidisciplinary approach that includes careful monitoring of renal function, blood pressure, and fluid balance to mitigate potential complications for both the mother and the developing fetus [2].

Anesthesia management is crucial for pregnant women with CKD due to the complex interplay between renal dysfunction and perioperative considerations. CKD alters fluid dynamics, electrolyte balance, and drug metabolism, increasing the complexity of anesthesia administration. The choice of anesthesia techniques, such as general anesthesia, regional anesthesia (e.g., epidural or spinal anesthesia), or local anesthesia, must be carefully tailored to the patient's renal function and the specific surgical or obstetric procedure [3]. Additionally, CKD patients are at higher risk for cardiovascular complications, including hypertension and fluid overload, which require vigilant monitoring and management during the perioperative period. Effective anesthesia management ensures not only maternal comfort and safety during labor and delivery but also optimizes fetal outcomes by minimizing the risks associated with maternal complications [4].

This review aims to comprehensively examine the current knowledge and challenges of anesthesia management in antenatal care for women with CKD. The review seeks to evaluate the efficacy and safety of various anesthesia techniques in this patient population by synthesizing existing literature and clinical practices. It will discuss perioperative considerations for CKD patients, including pre-operative assessment protocols, intraoperative monitoring strategies, and postoperative care guidelines. Furthermore, the review will analyze clinical outcomes associated with different anesthesia approaches, considering the impact of CKD on maternal and fetal health. By identifying gaps in current research and proposing future directions, the review aims to enhance anesthesia protocols and improve the overall care standards for pregnant women with CKD undergoing surgical procedures or labor management.

## Review

Pathophysiology of CKD in pregnancy

Changes in Renal Function During Pregnancy

The key changes in renal function during pregnancy include a significant increase in glomerular filtration rate (GFR), which rises by 40%-50%, leading to decreased serum creatinine levels. This increase in GFR is one of the earliest and most dramatic alterations in renal physiology during pregnancy. Additionally, renal plasma flow (RPF) increases by 50%-80% compared to non-pregnant levels. The increase in GFR and RPF is attributed to vasodilation and volume expansion during pregnancy [5]. Pregnancy also brings about changes in tubular function. There is decreased reabsorption of substances like glucose, uric acid, amino acids, and β-2-microglobulin, leading to increased excretion. Due to this decreased tubular reabsorption, pregnant women may exhibit glycosuria even at normal blood glucose levels. Serum uric acid levels typically fall due to increased filtration and decreased reabsorption. Furthermore, the kidneys increase in size by about 30% during pregnancy, and physiologic hydronephrosis, more commonly affecting the right kidney, occurs in up to 80% of pregnant women [6]. Understanding these normal physiological adaptations is crucial for interpreting laboratory values and managing kidney disease in pregnant women. The increased GFR and RPF can mask the severity of underlying CKD early in pregnancy, making monitoring renal function closely throughout the pregnancy essential. Additionally, the altered tubular function and anatomical changes can affect the interpretation of laboratory tests and the management of kidney disease in pregnant women [7].

Impact of CKD on Maternal and Fetal Health

**Figure 1:**
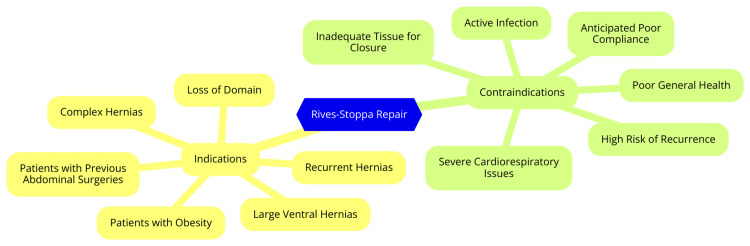
Impact of CKD on maternal and fetal health CKD - chronic kidney disease Image Credit: Dr. Angan Ghosh

CKD can significantly impact the health of both the mother and the fetus during pregnancy. The severity of CKD, as measured by GFR and proteinuria, is the main determinant of maternal and fetal outcomes. Maternal risks associated with CKD in pregnancy include an increased likelihood of developing hypertension (25%) and proteinuria (50%) during pregnancy. Women with CKD also face a higher risk of preterm delivery, especially with advanced CKD (stages 3-5). Additionally, there is an accelerated decline in maternal renal function, particularly in CKD stages 3b-5. Pregnant women with CKD are at an increased risk of pre-eclampsia, especially those with uncontrolled hypertension or nephrotic-range proteinuria. Anemia and gestational diabetes are also more common in this population, particularly in women on steroids, of Asian or African descent, or who are overweight [8]. On the fetal side, CKD in pregnancy is associated with a higher risk of fetal growth restriction, with the risk being greater in CKD stages 4-5. There is also an increased risk of preterm birth, with the risk being higher in advanced CKD. Consequently, neonatal unit admissions are more common, and there is an increased risk of stillbirth and neonatal mortality [9]. The likelihood of adverse outcomes predominantly depends on baseline excretory renal function, hypertension, proteinuria, and, to a lesser extent, the underlying cause of CKD. However, even women with preserved excretory renal function (CKD stages 1-2) have a higher risk of complications compared to the general obstetric population [10]. The impact of CKD on maternal and fetal health is illustrated in Figure 1.

Anesthesia considerations for women with CKD

Pre-operative Assessment and Risk Stratification

To ensure safe and effective anesthesia management, preoperative assessment and risk stratification are essential for patients with CKD. The initial step is to determine the stage of CKD and its underlying etiology, which aids in evaluating renal function, electrolyte balance, and acid-base status. It is also crucial to assess the need for dialysis and schedule it appropriately. Identifying and managing comorbidities such as hypertension, diabetes, and cardiovascular disease is vital [11]. Risk stratification plays a critical role in the preoperative assessment. Patients with end-stage renal disease (ESRD) have a higher mortality rate in the perioperative setting compared to those without ESRD. Predictive model scores can be utilized to stratify risk for acute kidney injury (AKI), major adverse cardiac events, or other serious complications. Cardiovascular disease remains the most common cause of death in ESRD patients, making meticulous risk stratification essential, even in the absence of traditional risk factors [12]. Preoperative optimization is also important. Adjustments should be made to the dosages of really excreted drugs to avoid adverse effects. It is crucial to optimize fluid, electrolyte, and acid-base balance to prevent complications. For patients with volume overload, preoperative dialysis may be necessary. Nephrologists should be involved in the care of transplant recipients to ensure optimal management. Emergency surgery and advanced CKD carry higher surgical risks, necessitating a multidisciplinary approach to address these risks and provide individualized counseling to high-risk patients with advanced CKD [13].

Choice of Anesthesia Techniques

When choosing the anesthetic technique for women with CKD during pregnancy, several factors must be considered to ensure both maternal and fetal safety. General anesthesia may sometimes be necessary but requires meticulous planning and execution. Induction agents should be titrated cautiously due to altered pharmacokinetics in CKD patients. Succinylcholine should be avoided because of the risk of hyperkalemia, and non-depolarizing neuromuscular blocking drugs should be used with caution as their effects may be prolonged. Careful fluid balance and electrolyte monitoring are essential during general anesthesia [14]. Regional anesthesia techniques, such as spinal and epidural anesthesia, are often preferred for women with CKD. Spinal anesthesia is considered a safe and preferred technique for cesarean delivery in CKD parturients. Epidural anesthesia can also be used cautiously, but the risk of dural puncture and hypotension should be considered. Epidural anesthesia should be avoided in patients with severe coagulopathy or thrombocytopenia. When administering regional anesthesia, it is crucial to monitor for signs of local anesthetic toxicity [15]. Local anesthesia can be used for minor procedures, such as inserting central venous catheters or arterial lines. The dosage should be adjusted based on renal function to avoid toxicity. Vasoconstrictors like epinephrine should be avoided in patients with severe cardiovascular disease. When choosing the anesthetic technique, several factors should be considered, including the severity of CKD and associated comorbidities, the urgency and type of surgery, patient preference, contraindications to specific techniques, and the availability of resources and expertise [16].

Monitoring and Management of Anesthesia During Pregnancy

Fetal monitoring is a critical aspect of anesthetic management during pregnancy. Intraoperative electronic fetal heart rate monitoring should be performed before and after the procedure if the fetus is viable. While continuous fetal monitoring is often possible, it may be impractical in emergent situations. Interpreting fetal monitoring data requires expertise, as misinterpretations could lead to unsafe interventions [14]. The selection of anesthetic techniques is crucial. Regional anesthesia techniques, such as spinal and epidural anesthesia, are preferred to minimize systemic exposure. In some cases, general anesthesia may be necessary, but it requires meticulous planning and execution. Neuraxial anesthesia is favored, if feasible, but the increased engorgement of epidural veins and reduced epidural space volume in pregnancy can lead to an increased spread of local anesthetics and a higher risk of bloody tap [17]. Intraoperative management necessitates careful attention to fluid balance, medication dosing, and maternal-fetal well-being. Fluid balance must be meticulously managed to avoid over or underhydration. Medications should be titrated cautiously due to altered pharmacokinetics in pregnancy. Maintaining adequate uterine perfusion and fetal oxygenation is crucial, particularly if the fetus is compromised. Avoiding fetal respiratory acidosis and using the lowest possible pressure pneumoperitoneum are also important considerations [18]. Monitoring during anesthesia in pregnancy also requires special attention. The risk of difficult intubation and mask ventilation is increased in pregnancy, so video laryngoscopy should be utilized whenever available, with advanced airway devices readily accessible. Downsized endotracheal tubes are recommended, but nasal intubation is not advised. Additionally, the sedative effects of anesthetics may be observed at lower doses than in non-pregnant patients [19].

Special considerations and challenges

Management of Fluid Balance and Electrolytes

Managing fluid balance and electrolytes in patients with CKD is crucial due to the high risk of cardiovascular morbidity and mortality associated with fluid overload. For hemodialysis patients, optimal fluid and sodium imbalance management involves adjusting salt and fluid removal through dialysis and restricting salt intake and fluid gain between dialysis sessions. The "dry weight" approach aims to restore salt and water homeostasis, but recent studies suggest that aggressive fluid removal during dialysis can induce excessive hemodynamic stress and potential organ damage. Fluid status assessment relies on clinical evaluation, non-invasive instrumental tools, cardiac biomarkers, and algorithmic and sodium modeling to estimate mass transfer [20]. Patients on peritoneal dialysis (PD) undergo daily treatments, allowing for more frequent fluid removal than hemodialysis patients. PD patients are encouraged to monitor their fluid intake and the amount of fluid removed in their dialysis exchanges to avoid complications such as swelling, high blood pressure, and shortness of breath [21]. For patients in later stages of CKD, fluid restriction is necessary to prevent fluid buildup and its associated complications, such as high blood pressure and shortness of breath. Fluid restriction may vary for each patient based on weight gain between treatments, urine output, and swelling. In CKD, sodium retention occurs due to impaired sodium excretion and the relationship between GFR, sodium excretion, and extracellular volume. CKD patients exhibit an adaptive response to a reduced nephron number by increasing sodium excretion, which can lead to subclinical volume expansion and elevated blood pressure [22]. Renal patients are predisposed to both dehydration and overhydration, which must be carefully managed to avoid complications such as renal hypoperfusion and additional ischemic renal injury. Fluid balance in polyuric renal disease is maintained by compensatory polydipsia, but insufficient water consumption can lead to dehydration. Determining fluid status in CKD patients is challenging due to the complexity of regulatory systems and the lack of standardized technologies to determine fluid distribution adequately. A multimodal clinical approach is proposed for volume management in CKD, including clinical observation, technological methods, and consideration of biomarkers like VEGF-C [23].

Pharmacokinetic Alterations and Drug Dosing

Pharmacokinetic alterations in patients with CKD significantly impact drug dosing and efficacy. Absorption can be poorly quantified and may increase or decrease, leading to changes in bioavailability. The volume of distribution may remain unchanged or increase, affecting the initial dose required. Metabolism is generally decreased, with clearance by cytochrome P450 enzymes (CYPs) being affected. Renal excretion is decreased but relatively stable compared to AKI. Elimination generally decreases due to reduced renal clearance, though kidney replacement therapies can increase drug clearance [24]. In CKD, initial dosing is generally the same as in patients with normal kidney function, but higher loading doses may be needed to achieve therapeutic concentrations quickly. Maintenance dosing adjustments include dose reduction, lengthening the dosing interval, or both to maintain more constant drug concentrations while minimizing toxicities [25]. In AKI, absorption is also poorly quantified and may decrease. The volume of distribution may remain unchanged or increase. Metabolism is poorly quantified, and clearance by CYP3A4/5 may decrease. Excretion is increased due to drug removal by kidney replacement therapies, but the extent depends on the drug and the therapy. Initial dosing is often the same as in patients with normal kidney function, but higher loading doses may be used to achieve therapeutic concentrations rapidly. Maintenance dosing adjustments are more dynamic and require frequent monitoring due to the rapidly changing kidney function [24]. Special considerations in CKD include avoiding medications with toxic metabolites, using the least nephrotoxic agents, and considering alternative medications if potential drug interactions exist. In AKI, using higher initial doses and adjusting maintenance dosing more frequently are crucial to account for the rapidly changing kidney function [26].

Impact of CKD on Anesthesia Recovery and Postoperative Care

The impact of CKD on anesthesia recovery and postoperative care is significant and requires careful management. Clearance of anesthetic drugs is reduced in CKD patients, leading to prolonged recovery times. The effects of neuromuscular blocking agents like rocuronium become less predictable and may last longer in renal failure. Careful titration of anesthetic drugs is required to avoid accumulation and toxicity [4]. Postoperative care for CKD patients is equally challenging. Achieving fluid balance is difficult and must be monitored closely to avoid overhydration or dehydration. Electrolyte abnormalities, such as hyperkalemia, are common and must be managed promptly. CKD patients are at higher risk of postoperative AKI, which is associated with worse outcomes. Delayed gastric emptying and neuropathy increase the risk of aspiration during the postoperative period. Impaired wound healing due to poor nutrition and vascular disease is also a concern [27]. Overall, the anesthetic management and postoperative care of CKD patients require a multidisciplinary approach. This involves closely monitoring and titrating fluids, electrolytes, and medications to minimize complications and optimize recovery. Early detection of CKD and careful preoperative optimization are crucial for improving outcomes in this high-risk population. Anesthesiologists must recognize the unique challenges of CKD and adapt their management strategies accordingly to ensure the best possible outcomes for their patients [28].

Clinical outcomes and complications

Maternal Outcomes

Anesthesia management in antenatal care for women with CKD requires a comprehensive approach to minimize maternal risks. Pregnancy in CKD patients is associated with poor maternal outcomes that correlate strongly with the stage of CKD and the amount of proteinuria. Complications such as acute renal failure, hyperkalemia, volume overload, and infections are more common in CKD patients undergoing surgery. Careful fluid management is crucial to avoid overhydration or dehydration, which can destabilize the cardiovascular system. Maintaining normothermia and avoiding acidosis is important to prevent maternal complications [29]. General anesthesia should be used cautiously in CKD patients, with awareness of the increased risks of hyperkalemia and prolonged drug effects. Spinal anesthesia is generally preferred for cesarean delivery as it avoids the risks associated with general anesthesia. Altered drug metabolism in CKD patients requires careful titration of anesthetic agents to avoid accumulation and toxicity. A multidisciplinary approach involving nephrologists, anesthesiologists, obstetricians, and other healthcare providers is necessary to ensure successful anesthesia management and minimize maternal risks. Close monitoring and individualized care plans are essential to address the unique challenges posed by CKD in pregnant patients [30].

Fetal and Neonatal Outcomes

Fetal and neonatal outcomes for women with CKD are a significant concern. Neonates of mothers with CKD face increased risks of preterm birth, fetal growth restriction, small for gestational age infants, neonatal mortality, stillbirths, and low birth weight. These risks are particularly pronounced: preterm birth occurs in 20-50% of cases, fetal growth restriction is five times more common, neonatal mortality is five times higher, stillbirths are nine times more likely, and low birth weight is five times more common in these pregnancies [31]. To mitigate these risks, fetal surveillance is crucial. This includes first and second-trimester screening, bi-weekly growth scans after 28-30 weeks with Doppler studies to detect fetal growth restriction, and cardiotocography for fetal evaluation. Serum human chorionic gonadotropin levels may be elevated in advanced CKD, necessitating alternative prenatal testing. The delivery timing is also critical and should be determined by obstetric indications, considering renal factors such as deteriorating renal function, hypoalbuminemia, and pulmonary edema. Elective delivery is indicated if labor does not occur by 39-40 weeks, and earlier delivery may be necessary in cases of uncontrolled hypertension, severe fetal growth restriction, or an abnormal fetal biophysical profile [32].

Long-Term Implications for Both Mother and Child

CKD in pregnancy carries significant long-term implications for both the mother and child. For the mother, the degree of renal dysfunction strongly correlates with the risk of poor pregnancy outcomes. Women with moderate to severe CKD (stages 3-5) face the highest risks, including accelerated decline in renal function, uncontrolled hypertension, heavy proteinuria, and recurrent urinary tract infections. These factors independently and cumulatively contribute to adverse maternal outcomes, with those with severe renal impairment experiencing challenges in conceiving, higher rates of miscarriage, and overall poorer pregnancy outcomes. Furthermore, gestational hypertension and preeclampsia in these patients elevate the long-term risk of developing CKD and end-stage kidney disease [33]. For the fetus and neonate, the risks are also considerable. Intrauterine growth retardation and preterm delivery are more prevalent among mothers with advanced CKD, with the risk escalating as the maternal CKD stage worsens. Babies born to mothers with CKD may also have an increased likelihood of inheriting congenital kidney abnormalities, necessitating vigilant screening. Overall, the severity of maternal kidney disease emerges as the principal determinant of outcomes for both mother and child, underscoring the critical need for meticulous multidisciplinary management to optimize their health [34].

Evidence-based practices and guidelines

Current Recommendations From Obstetric and Anesthesia Societies

The anesthetic management of women with CKD undergoing antenatal care and cesarean delivery demands a comprehensive, multidisciplinary approach aimed at minimizing maternal and fetal risks. Spinal anesthesia is generally preferred for cesarean delivery in CKD patients due to its avoidance of the risks associated with general anesthesia. Regional anesthesia techniques, such as epidural or combined spinal epidural, are also viable options, offering the advantage of precise anesthetic titration. General anesthesia should be used cautiously, considering the heightened risks of hyperkalemia and prolonged drug effects [35]. Careful fluid management is crucial to maintain optimal hydration and stabilize the cardiovascular system. Medications must be dosed cautiously, tailored to the patient's renal function, and closely monitored for potential adverse effects. Maintaining normothermia and preventing acidosis are pivotal to minimizing complications. Postoperative care should include vigilant monitoring of renal function, electrolytes, and fluid balance to ensure optimal recovery. Women with CKD benefit from expert, multidisciplinary pre-pregnancy counseling to discuss the increased risks of complications and optimize management strategies. A planned early postpartum renal review is essential to monitor and address any potential exacerbation of kidney function after delivery [36].

Case Studies and Clinical Trials Evaluating Anesthesia Management in CKD Patients

Case studies and clinical trials evaluating anesthesia management in CKD patients are crucial for understanding the complexities and risks associated with anesthesia in this vulnerable population. A retrospective study involving nine parturients with CKD undergoing elective cesarean delivery demonstrated the safe administration of spinal anesthesia with minimal side effects. This study underscored the importance of meticulous fluid management and tailored medication dosing based on renal function to mitigate risks like hypotension and electrolyte imbalances [3]. The management of CKD patients necessitates a thorough preoperative assessment, including comprehensive history-taking, physical examination, and laboratory tests to evaluate renal function, fluid status, and potential for perioperative complications. Preoperative dialysis may be required to achieve optimal volume status and electrolyte balance. A case report on a CKD patient undergoing exploratory laparotomy highlighted the efficacy of regional anesthesia techniques, such as bupivacaine, in managing pain effectively while minimizing systemic exposure. The report also emphasized the critical need for vigilant fluid management and continuous monitoring of hemodynamic stability during and after surgery [13]. General anesthesia should be approached cautiously in CKD patients due to the heightened risks of complications like hyperkalemia and prolonged drug effects. Regional anesthesia methods, such as epidural or spinal anesthesia, are generally preferred to limit systemic exposure and reduce the incidence of adverse outcomes. These studies and case reports underscore the importance of adopting a multidisciplinary approach to anesthesia management in CKD patients, focusing on thorough preoperative assessment, precise intraoperative care, and vigilant postoperative monitoring to optimize outcomes [3].

## Conclusions

In conclusion, effective anesthesia management is paramount for ensuring safe and successful outcomes in pregnant women with CKD. The complexities introduced by CKD, including altered renal function, fluid dynamics, and increased cardiovascular risks, necessitate a tailored approach to anesthesia selection and perioperative care. This review has highlighted the importance of meticulous pre-operative assessment, careful intraoperative monitoring, and appropriate postoperative management strategies to mitigate risks and optimize maternal and fetal health outcomes. By synthesizing current evidence and addressing clinical challenges, this review underscores the need for interdisciplinary collaboration and ongoing research to refine anesthesia protocols for this vulnerable patient population. Ultimately, advancing our understanding and implementation of anesthesia management in antenatal care for CKD patients will contribute to improved clinical practices and better overall care standards in obstetric anesthesia.
